# Elucidation of the core betalain biosynthesis pathway in *Amaranthus tricolor*

**DOI:** 10.1038/s41598-021-85486-x

**Published:** 2021-03-17

**Authors:** Yu-Cheng Chang, Yi-Ching Chiu, Nai-Wen Tsao, Yuan-Lin Chou, Choon-Meng Tan, Yi-Hsuan Chiang, Pei-Chi Liao, Ya-Chien Lee, Li-Ching Hsieh, Sheng-Yang Wang, Jun-Yi Yang

**Affiliations:** 1 Institute of Biochemistry, National Chung Hsing University, 145 Xingda Road, Taichung, 40227 Taiwan; 2 Department of Forestry, National Chung Hsing University, Taichung, 402 Taiwan; 3 Institute of Genomics and Bioinformatics, National Chung Hsing University, Taichung, 402 Taiwan; 4 Institute of Biotechnology, National Chung Hsing University, Taichung, 402 Taiwan; 5 Advanced Plant Biotechnology Center, National Chung Hsing University, Taichung, 402 Taiwan

**Keywords:** Natural variation in plants, Plant biotechnology, Plant molecular biology, Secondary metabolism

## Abstract

*Amaranthus tricolor* L., a vegetable *Amaranthus* species, is an economically important crop containing large amounts of betalains. Betalains are natural antioxidants and can be classified into betacyanins and betaxanthins, with red and yellow colors, respectively. *A. tricolor* cultivars with varying betalain contents, leading to striking red to green coloration, have been commercially produced. However, the molecular differences underlying betalain biosynthesis in various cultivars of *A. tricolor* remain largely unknown. In this study, *A. tricolor* cultivars with different colors were chosen for comparative transcriptome analysis. The elevated expression of *AmCYP76AD1* in a red-leaf cultivar of *A. tricolor* was proposed to play a key role in producing red betalain pigments. The functions of *AmCYP76AD1*, *AmDODAα1*, *AmDODAα2*, and *AmcDOPA5GT* were also characterized through the heterologous engineering of betalain pigments in *Nicotiana benthamiana*. Moreover, high and low L-DOPA 4,5-dioxygenase activities of AmDODAα1 and AmDODAα2, respectively, were confirmed through in vitro enzymatic assays. Thus, comparative transcriptome analysis combined with functional and enzymatic studies allowed the construction of a core betalain biosynthesis pathway of *A. tricolor*. These results not only provide novel insights into betalain biosynthesis and evolution in *A. tricolor* but also provide a basal framework for examining genes related to betalain biosynthesis among different species of *Amaranthaceae*.

## Introduction

Betalains are classified into betacyanins and betaxanthins, which provide red-violet and yellow coloration, respectively^1^. Similar to anthocyanins, betalains exhibit antioxidant activity in the form of free-radical scavenging and accumulate in response to different stresses, such as UV-B radiation, high-intensity light, salinity, heat, and drought^2–5^. In addition to their potential roles in protecting plants against abiotic stresses, betalains play a role in defense against pathogenic fungi^6^. Moreover, as water-soluble natural pigments, betalains are widely used as food additives because of their health-promoting properties and color stability over a wide range of pH values^7,8^.

Betalains occur only in Caryophyllales and have never been detected in anthocyanin-producing plants^9,10^. Although the molecular basis for the mutual exclusion of betalains and anthocyanins is still unclear, breakthroughs in the identification of genes involved in betalain biosynthesis have shed light on the evolution of betalain pigmentation in Caryophyllales^9–12^. Unlike anthocyanins, which are derived from L-phenylalanine, betalains are synthesized from L-tyrosine^13,14^. Initially, L-tyrosine is hydroxylated to produce L-DOPA by tyrosinases encoded by *CYP76AD1* and its orthologs^15,16^. L-DOPA can be converted into betalamic acid by L-DOPA 4,5-dioxygenase encoded by *DODA,* and betalamic acid can then spontaneously condense with amino acids to form betaxanthins^1,12,17^. Alternatively, L-DOPA can be converted into *cyclo*-DOPA through the oxidase activity of CYP76AD1, and *cyclo*-DOPA can then spontaneously condense with betalamic acid to form betanidin^9,18^. Betanidin is further glycosylated by betanidin 5-*O*-glucosyl-transferase encoded by *B5GT* to form betanin, the most common betacyanin. Glycosylation can also occur on *cyclo*-DOPA, catalyzed by *cyclo*-DOPA 5-*O*-glucosyltransferase encoded by *cDOPA5GT*, to produce *cyclo*-DOPA-glucoside, which then spontaneously condenses with betalamic acid to form betanin^10,19^.

Phylogenetic analyses revealed that the *CYP76AD* and *DODA* genes, encoding key enzymes in the core biosynthetic pathway of betalains, are highly duplicated in Caryophyllales^10,20^. The *CYP76AD* gene lineage has undergone at least three duplication events, giving rise to three clades: CYP76Adα, CYP76Adβ and CYP76ADγ^9^. The CYP76Adα clade includes the *CYP76AD1* and *CYP76AD3* genes, whose products possess both the tyrosine hydroxylase and L-DOPA 4,5-dioxygenase activities required for L-DOPA and *cyclo*-DOPA formation, respectively^16^. The CYP76ADβ clade includes the *CYP76AD5*, *CYP76AD6*, and *CYP76AD15* genes, which possess only the tyrosine hydroxylase activity required for L-DOPA formation^16^. However, the functions of the genes in the CYP76ADγ clade in betalain biosynthesis have not been determined. Duplication events in the *DODA* gene lineage also gave rise to two major clades: DODAα and DODAβ. The function of DODAβ is unknown, but the evolution of L-DOPA 4,5-dioxygenase activity in betalain-producing plants was proposed to be led by DODAα^9^. Nevertheless, only one paralogous gene from the DODAα clade shows high L-DOPA 4,5-dioxygenase activity in each species, and others exhibit barely detectable activity^12,21^.

An increased understanding of the betalain biosynthesis pathway has facilitated the metabolic engineering of betalains, providing new sources for basic research studies and commercial applications^22,23^. For example, the fluorescent betaxanthins produced by the expression of *MjDODAα1* in yeast were used as chemical biosensors to reveal the tyrosine hydroxylase activity of BvCYP76AD1^15^. The production of semisynthetic betaxanthins by spontaneous condensation between fluorescent betalamic acid and the amino groups of proteins provides an alternative method for labeling proteins for multiple biological applications^24^. Fine-turning the content ratio of betacyanins and betaxanthins via the differential expression of *BvCYP76AD1* and *BvCYP76AD6* in non-Caryophyllales makes it possible to create a range of color patterns in the background of anthocyanin-producing plants^6^. The heterologous production of betalains via the coexpression of *BvCYP76AD1*, *BvDODAα1*, and *MjcDOPA5GT* in tobacco enhances the resistance of transgenic plants to *Botrytis cinereal* infection^6^. In addition, betanin rice generated through the coexpression of *meloS*, *BvCYP76AD1*, and *BvDODAα1* in rice endosperm shows higher antioxidant activity and can be provided as a functional food^25^.

Amaranth species are economically important crops containing large amounts of betalains. They can be classified into three categories: vegetable *Amaranthus*, grain *Amaranthus*, and weed *Amaranthus* species^26^. *Amaranthus tricolor* L., a vegetable *Amaranthus* species, is widely distributed in warm and tropical regions and is cultivated as a leafy vegetable. As rich in natural antioxidants, *A. tricolor* is able to tolerate abiotic stresses and has been used as a traditional Chinese medicinal herb for the treatment of eruptive fever, pain, sore throat, dysentery, anemia, bronchitis, colic, etc^27^. *A. tricolor* cultivars with various contents of betacyanins and betaxanthins, resulting in striking colors ranging from red to green, have been commercially produced^27,28^. However, the molecular basis underlying betalain biosynthesis in *A. tricolor* remains largely unknown. Recently, virus-induced gene silencing was applied to elucidate the function of *CYP76AD1* in producing betalain pigments in *A. tricolor*^29^. In addition, a comparative analysis of a transcriptome database constructed from different leaf samples of *A. tricolor* cv. Dahong was performed to construct a putative metabolic pathway of betalains in *A. tricolor*^30^. Candidate genes encoding enzymes catalyzing the formation of L-DOPA, *cyclo*-DOPA, betalamic acid, *cyclo*-DOPA-glucoside, and betanin were obtained from a transcriptome database and showed higher expression levels in red areas of *A. tricolor* leaves than in in green areas^30^. However, functional characterization and enzyme activity analyses are still needed to elucidate the roles of these candidate genes in the betalain biosynthesis pathway of *A. tricolor*.

In this study, *A. tricolor* cultivars with different colors were chosen for comparative transcriptome analysis. The key gene showing elevated expression in a red-leaf cultivar of *A. tricolor* was identified, and the results indicated that the dual activities of tyrosine hydroxylase and L-DOPA oxidase are important for producing red betalain pigments in *A. tricolor*. The core betalain biosynthesis pathway of *A. tricolor* was further constructed based on the functional characterization of betalain biosynthesis genes through the heterologous engineering of betalain pigments in *Nicotiana benthamiana* and in vitro enzymatic assays of L-DOPA 4,5-dioxygenase activities. These results provide novel insights into betalain biosynthesis and evolution in *A. tricolor*.

## Results

### *AmCYP76AD1 *is highly expressed in a red-leaf cultivar of* A. tricolor*

**Figure 1:**
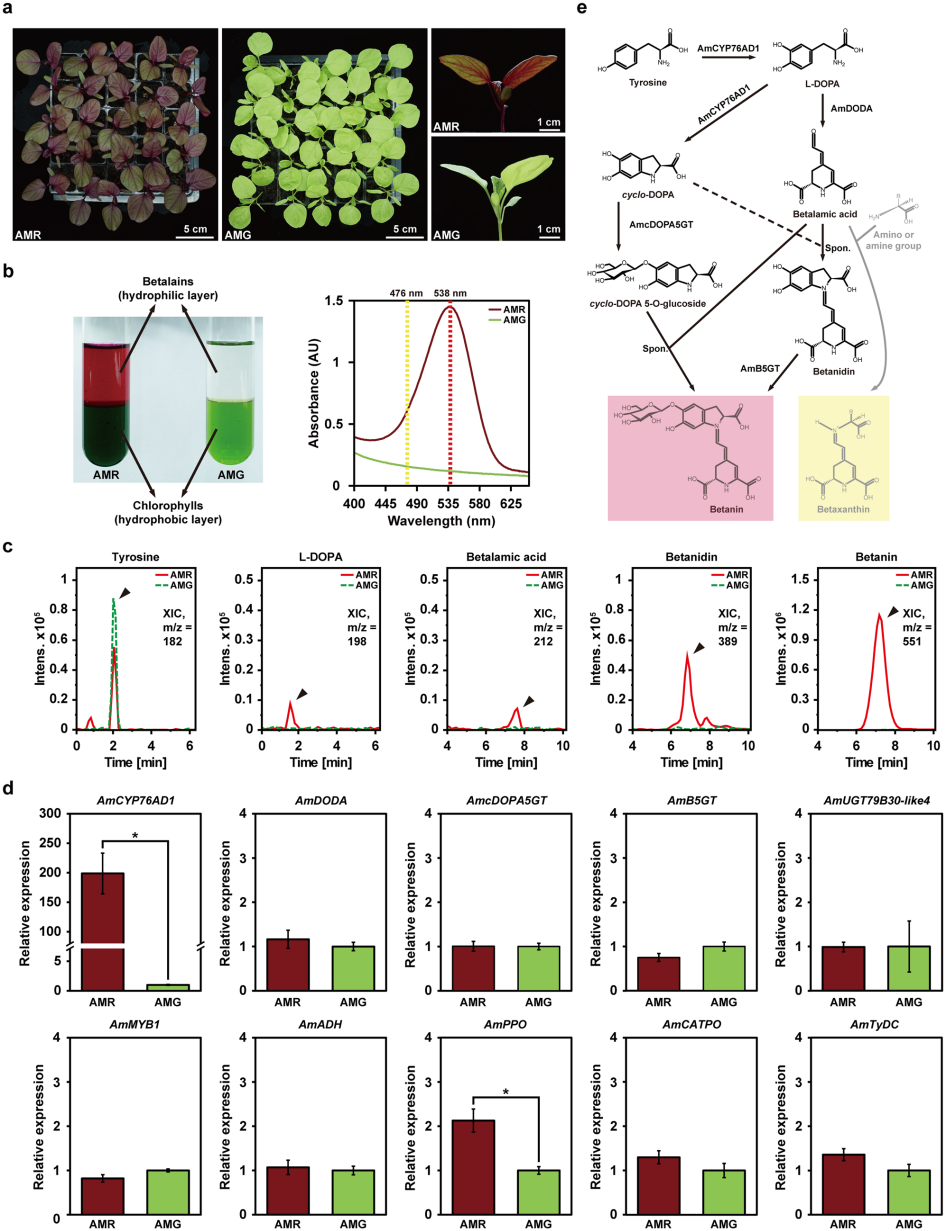
Identification of *AmCYP76AD1* as a key element required for betalain pigment production in *Amaranthus tricolor*. (**a**) The leaf-color phenotypes of the red-leaf cultivar (AMR) and green-leaf cultivar (AMG) of three-week-old *A. tricolor*. (**b**) Extraction of chlorophyll pigments (hydrophobic layer) and betalain pigments (hydrophilic layer) from three-week-old leaves of AMR and AMG (left panel). Absorbance spectra of the extracted betalain pigments from AMR and AMG (right panel). The absorbance at 538 nm for betacyanins is indicated with a red dashed line, and the absorbance at 476 nm for betaxanthins is indicated with a yellow dashed line. (**c**) Liquid chromatography-tandem mass spectrometry (LC–MS/MS) analysis of three-week-old leaves of AMR and AMG. Shown are extracted ion chromatograms (XICs) of masses corresponding to tyrosine (m/z = 182), *L*-DOPA (m/z = 198), betalamic acid (m/z = 212), betanidin (m/z = 389), and betanin (m/z = 551). Time, retention time (min). (**d**) Expression levels of genes related to the betalain biosynthesis pathway in AMR and AMG analyzed by qRT-PCR. Statistically significant differences were determined using Student’s *t*-test (*P < 0.01 for AMR vs. AMG). (**e**) Putative core betalain biosynthesis pathway in *A. tricolor*. *Am*, *Amaranthus tricolor*; *CYP76AD1*, *cytochrome P450 76AD1*; *DODA*, *DOPA-4,5-dioxygenase*; *cDOPA5GT*, *cyclo-DOPA 5-O-glucosyltransferase*; *B5GT*, *betanidin-5-O-glucosyltransferase*; *UGT79B30-like 4*, *UDP-glucose glucosyltransferase 79B30-like 4*; *ADH*, *arogenate dehydrogenase*; *PPO*, *polyphenol oxidase*; *CATPO*, *catalase-phenol oxidase*; *TyDC*, *tyrosine decarboxylase*.

**Figure 2:**
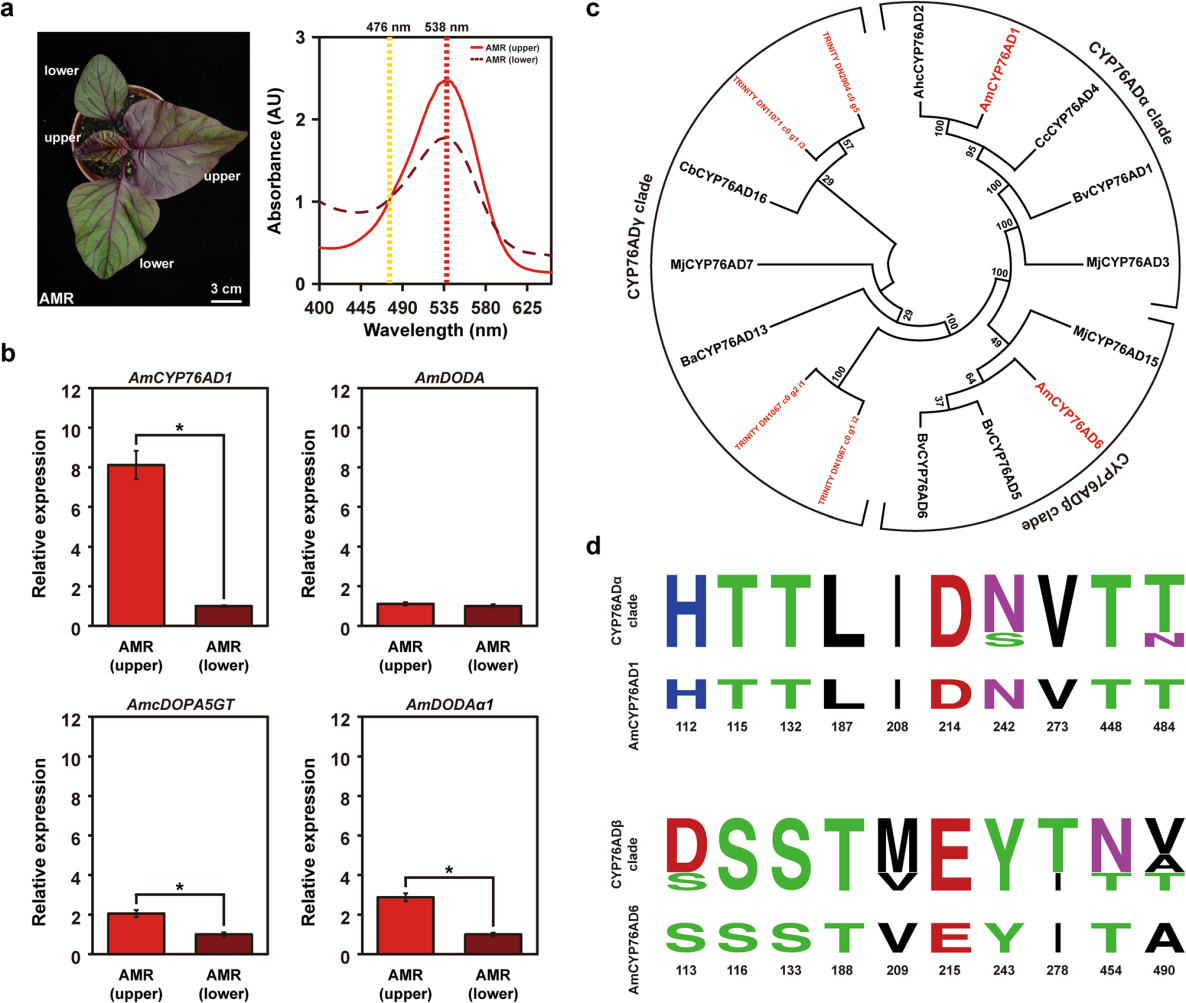
Expression pattern, phylogenetic reconstruction, and LOGO analysis of *AmCYP76AD1*. (**a**) The leaf color phenotypes and betalain absorbance spectra of the upper and lower leaves of the red-leaf cultivar (AMR) of four-week-old *A. tricolor*. (**b**) Expression levels of core betalain biosynthesis genes in the upper and lower leaves of four-week-old AMR plants analyzed by qRT-PCR. Statistically significant differences were determined using Student’s *t*-test (*P < 0.01 for the upper leaves of AMR vs. the lower leaves of AMR). (**c**) Phylogenetic tree of CYP76AD homologues. The species, families, and accession numbers of CYP76AD homologues are available in Supplementary Table S6. (**d**) Proportional LOGO plots of selected amino acids identified by Brockington et al^9^ were generated based on the CYP76ADα and CYP76ADβ homologues listed in (**c**). Positions are numbered according to the residues of AmCYP76AD1 and AmCYP76AD6.

*A. tricolor* cultivars are important leafy vegetables that display leaf colors ranging from red to green depending on the betalain content^28^. To elucidate the genetic factors that influence betalain pigment contents in red- and green-leaf cultivars of *A. tricolor* (hereafter referred to as AMR and AMG, respectively) (Fig. 1a–c, Supplementary Fig. S1a, b), specific primer pairs were designed based on available sequence information from the NCBI database or previously published studies to selectively examine the transcript levels of genes related to the betalain biosynthesis pathway by qRT-PCR (Supplementary Table S1). Among them, *AmADH*, *AmCYP76AD1*, *AmDODA*, *AmcDOPA5GT*, *AmB5GT*, *and AmUGT79B30-like 4* genes encoding for the putative enzymes were thought to be directly involved in the betalain biosynthesis^30^. *AmMYB1*, encoding an R2R3-type transcription factor, was proposed to participate in the regulation of betalain biosynthesis^10,30^. *AmPPO* and *AmCATPO* genes, encoding tyrosinases, were considered for their roles in conversion of L-DOPA to *cyclo*-DOPA^1,30^. *AmTyDC*, encoding a tyrosine decarboxylase, was proposed to participate in the degradation of betalain^1,30^. In 3-week-old *A. tricolor*, *AmCYP76AD1* and *AmPPO* showed higher expression levels in AMR than in AMG (Fig. 1d). Notably, only *AmCYP76AD1* exhibited a highly differential expression pattern, showing an ~ 200-fold difference between AMR and AMG. In contrast, *AmDODA*, *AmcDOPA5GT*, *AmB5GT*, *AmUGT79B30-like 4*, *AmMYB1*, *AmADH*, *AmCATPO*, and *AmTyDC* did not show a significant differential expression pattern between AMR and AMG (Fig. 1d). The highly differential expression pattern of *AmCYP76AD1* between AMR and AMG was also observed in 4-week-old *A. tricolor* (Supplementary Fig. S1c). Moreover, as a key element in the initiation of the betalain biosynthesis pathway, *AmCYP76AD1* displayed higher transcript levels in the upper leaves of AMR, which contained higher content of betalains than those in the lower leaves of AMR (Fig. 2a,b, Supplementary Fig. S2). Further phylogenetic reconstruction and LOGO analysis revealed that AmCYP76AD1 belongs to the CYP76Adα clade (Fig. 2c,d), whose members possess both the tyrosine hydroxylase and L-DOPA oxidase activities required for L-DOPA and *cyclo*-DOPA formation, respectively (Fig. 1e). These results suggest that the elevated expression of *AmCYP76AD1* is necessary for betalain pigment accumulation, which leads to an obvious red-violet color in the leaves and stems of AMR, but not in those of AMG.

### *AmDODA* exhibits a marginal level of L-DOPA 4,5-dioxygenase activity

**Figure 3:**
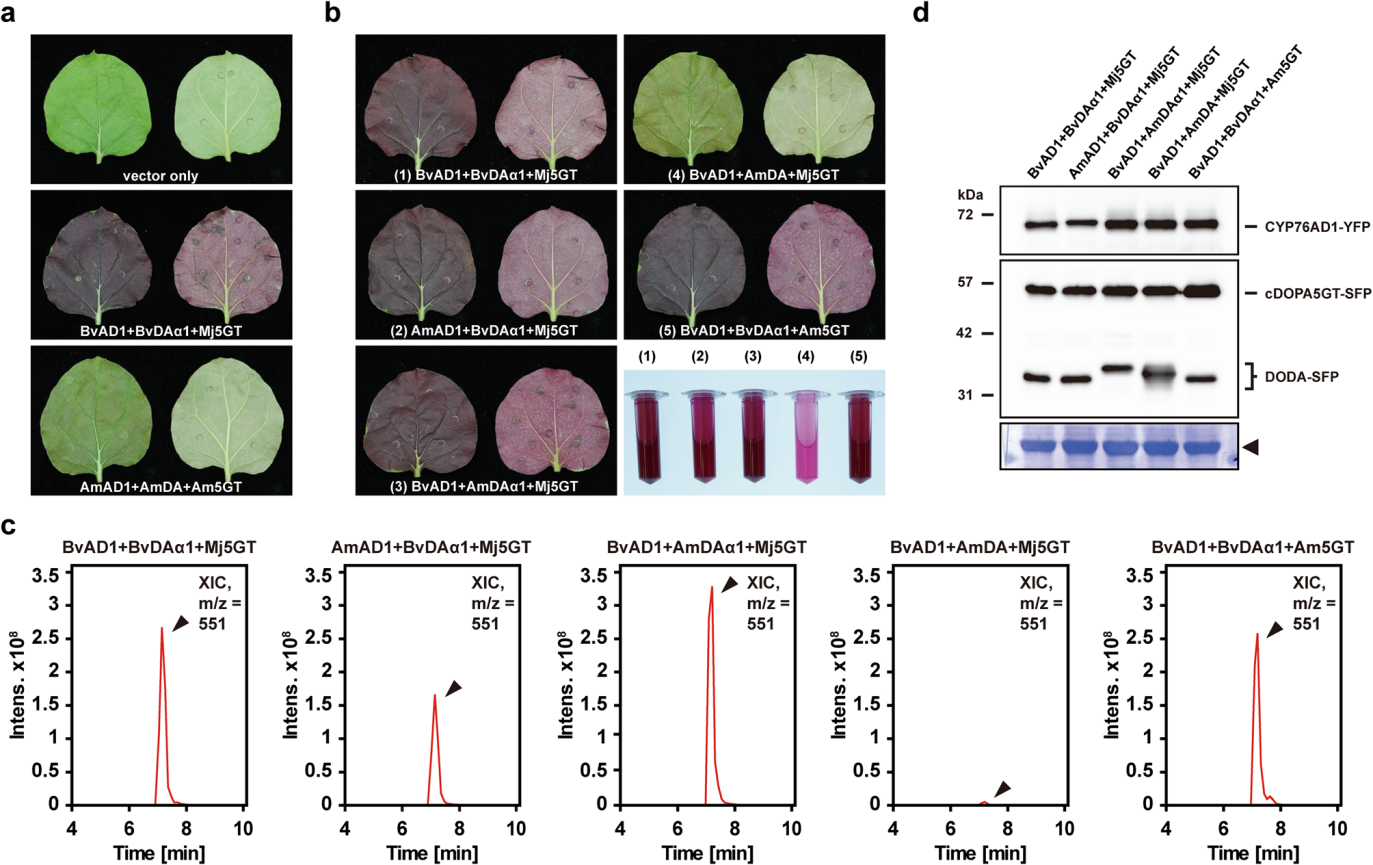
Functional characterization of the enzyme activities of AmCYP76AD1, AmDODA, and AmcDOPA5GT by agroinfiltration for the heterologous engineering of betalain pigments in *Nicotiana benthamiana*. (**a**, **b**) *N. benthamiana* leaves coinfiltrated with *Agrobacterium* harboring plasmids for the expression of *BvCYP76AD1-YFP* (*BvAD1*), *BvDODAα1-SFP* (*BvDAα1*), *MjcDOPA5GT-SFP* (*Mj5GT*), *AmCYP76AD1-YFP* (*AmAD1*), *AmDODA-SFP* (*AmDA*), and *AmcDOPA5GT-SFP* (*Am5GT*). Both the adaxial (left) and abaxial (right) sides of leaves are presented in each panel. Bottom right corner indicated the betalain pigments produced in *N. benthamiana*. (**c**) Extracted betalain pigments were examined for betanin content by LC–MS/MS analysis. Shown are XICs of masses corresponding to betanin (m/z = 551). Time, retention time (min). (**d**) Western blotting assays were conducted with antibodies against the YFP- or FLAG (SFP)-tag to examine the expression levels of YFP-tagged CYP76AD1 (upper panel), SFP-tagged DODA (middle panel), and SFP-tagged cDOPA5GT (middle panel). As a loading control, the large subunit of Rubisco visualized by Coomassie brilliant blue staining is indicated by the arrowhead (lower panel).

Although candidate transcripts related to betalain biosynthesis were identified previously in *A. tricolor*^30,31^, their functional and enzymatic activities have not yet been characterized. To functionally characterize the enzyme activities of AmCYP76AD1, AmDODA, and AmcDOPA5GT in the core pathway of betalain biosynthesis (Fig. 1e), *35S* promoter-driven cDNAs encoding C-terminal YFP- or FLAG (SFP)-tagged AmCYP76AD1, AmDODA, and AmcDOPA5GT were transiently coexpressed in *N. benthamiana* leaves by agroinfiltration. Upon expression, only a small amount of betalain pigment was produced in *N. benthamiana* leaves, which was barely detectable (Fig. 3a). In contrast, as a positive control, high production of betalain pigments with red-violet color was observed when the *Beta vulgaris* tyrosinase gene (*BvCYP76AD1*), the *B. vulgaris* L-DOPA 4,5-dioxygenase gene (*BvDODAα1*), and the *Mirabilis jalapa cyclo*-DOPA 5-*O*-glucosyltransferase gene (*MjcDOPA5GT*), were coexpressed in *N. benthamiana* leaves (Fig. 3a). To elucidate the *A. tricolor* genes responsible for the negligible activity of betalain synthesis in transient analysis, a series of coinfiltration assays were carried out by replacing the positive control genes individually with *AmCYP76AD1*, *AmDODA*, and *AmcDOPA5GT*. The replacements of *BvCYP76AD1* and *MjcDOPA5GT* by *AmCYP76AD1* and *AmcDOPA5GT*, respectively, resulted in high amounts of betalain pigment and betanin production in *N. benthamiana* leaves (Fig. 3b,c). However, *AmDODA* failed to replace the function of *BvDODAα1*. The coexpression of *BvCYP76AD1*, *AmDODA*, and *MjcDOPA5GT* only produced marginal levels of betalain pigments and betanin, which were barely detectable (Fig. 3b,c). Together with the comparable levels of proteins detected by western blotting (Fig. 3d, Supplementary Fig. S3), these results suggest that the L-DOPA 4,5-dioxygenase activity of AmDODA is very low compared to that of BvDODAα1.

### Two DODAα homologues are present in *A. tricolor*

**Figure 4:**
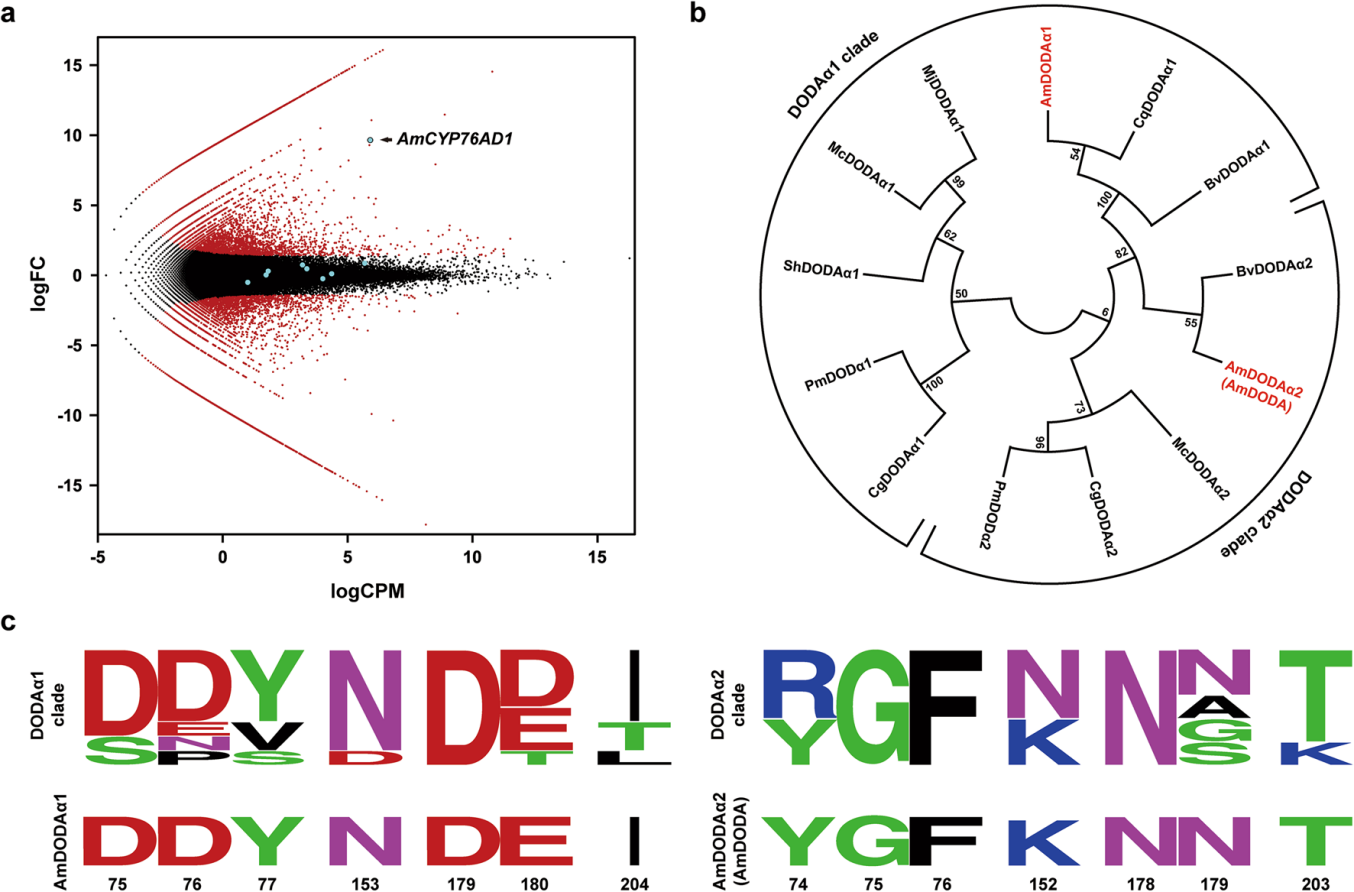
In silico analysis of relevant genes involved in the betalain biosynthesis pathway of *A. tricolor*. (**a**) The relative abundance of transcripts between AMR and AMG is presented in the MA plot (logCPM vs. logFC). Each dot presents a gene, and the relevant genes associated with betalain pigment synthesis are highlighted. (**b**) Phylogenetic tree of functionally characterized DODAα homologues. The species, families, and accession numbers of the DODAα homologues are available in Supplementary Table S6. (**c**) Proportional LOGO plots of seven functionally important residues identified by Bean et al. (2018)^21^ were generated based on the DODAα1 and DODAα2 homologues listed in (**b**). Positions are numbered according to the residues of AmDODAα1 and AmDODAα2.

Recently, a phylogenetic study of Caryophyllales suggested that at least two *DODAα* genes are present in betalain-pigmented species, including *Amaranthus hypochondriacus*^12^. To identify the DODAα homologue exhibiting a high level of L-DOPA 4,5-dioxygenase activity in *A. tricolor*, the RNA sequencing of aerial tissues derived from AMR and AMG plants was performed on the Illumina HiSeq 4000 platform. Two transcript libraries of AMR and AMG were built from the high-quality reads through de novo assembly and functional annotation (Supplementary Tables S2, S3). The relative abundance of transcripts between AMR and AMG was illustrated in an MA plot (Fig. 4a). In addition, the relevant genes involved in the synthesis of betalain pigments were identified through in silico analysis and further highlighted in the MA plot (Fig. 4a, Supplementary Table S4). As expected, only *AmCYP76AD1* was expressed at a significantly higher level in AMR than in AMG (Fig. 4a). These results suggest that AmCYP76AD1 is the key enzyme responsible for betalain pigment accumulation in AMR and that the loss of *AmCYP76AD1* expression in AMG results in the green color phenotype.

Additionally, two *DODAα* homologues, *AmDODAα1* and *AmDODAα2* (referred to as *AmDODA*), were recovered through in silico analysis (Supplementary Fig. S4, Table S4). This indicated that gene duplication has occurred at least once in the *DODAα* lineage of *A. tricolor*. A reduced phylogenetic tree of DODAα was further generated using AmDODAα1, AmDODAα2, and previously characterized DODAα homologues from *B. vulgaris*, *Carnegiea gigantea*, *Chenopodium quinoa, Mesembryanthemum crystallinum*, *M. jalap*a, *Parakeelya mirabilis*, and *Stegnosperma halimifolium* (Fig. 4b). Two clades, DODAα1 and DODAα2, were obtained, and each of them presented seven previously identified conserved residues that are functionally important for high and marginal activities of L-DOPA 4,5-dioxygenase, respectively (Fig. 4c). Among these sequences, AmDODAα1 belongs to the DODAα1 clade and contains seven residues (DDYNDEI) associated with high L-DOPA 4,5-dioxygenase activity; AmDODAα2 (AmDODA) belongs to the DODAα2 clade and contains seven residues (YGFKNNT) associated with marginal L-DOPA 4,5-dioxygenase activity. These results suggest that AmDODAα1 may exhibit the high level of L-DOPA 4,5-dioxygenase activity required for betalain pigment production in *A. tricolor*.

### AmDODAα1, but not AmDODAα2, exhibits a high level of L-DOPA 4,5-dioxygenase activity

As a key step in betalain biosynthesis, L-DOPA 4,5-dioxygenase can convert L-DOPA into betalamic acid, the basic structural unit of all betalains^1,32^. To functionally characterize the L-DOPA 4,5-dioxygenase activity of AmDODAα1, *AmDODAα1* was coexpressed with *BvCYP76AD1* and *MjcDOPA5GT* by agroinfiltration. As a result, high production of betalain pigments and betanin was observed when comparable amounts of proteins were expressed in *N. benthamiana* leaves (Fig. 3b–d, Supplementary Fig. S3). These results indicate that AmDODAα1, but not AmDODAα2, exhibits a high level of L-DOPA 4,5-dioxygenase activity, similar to that of BvDODAα1.

**Figure 5:**
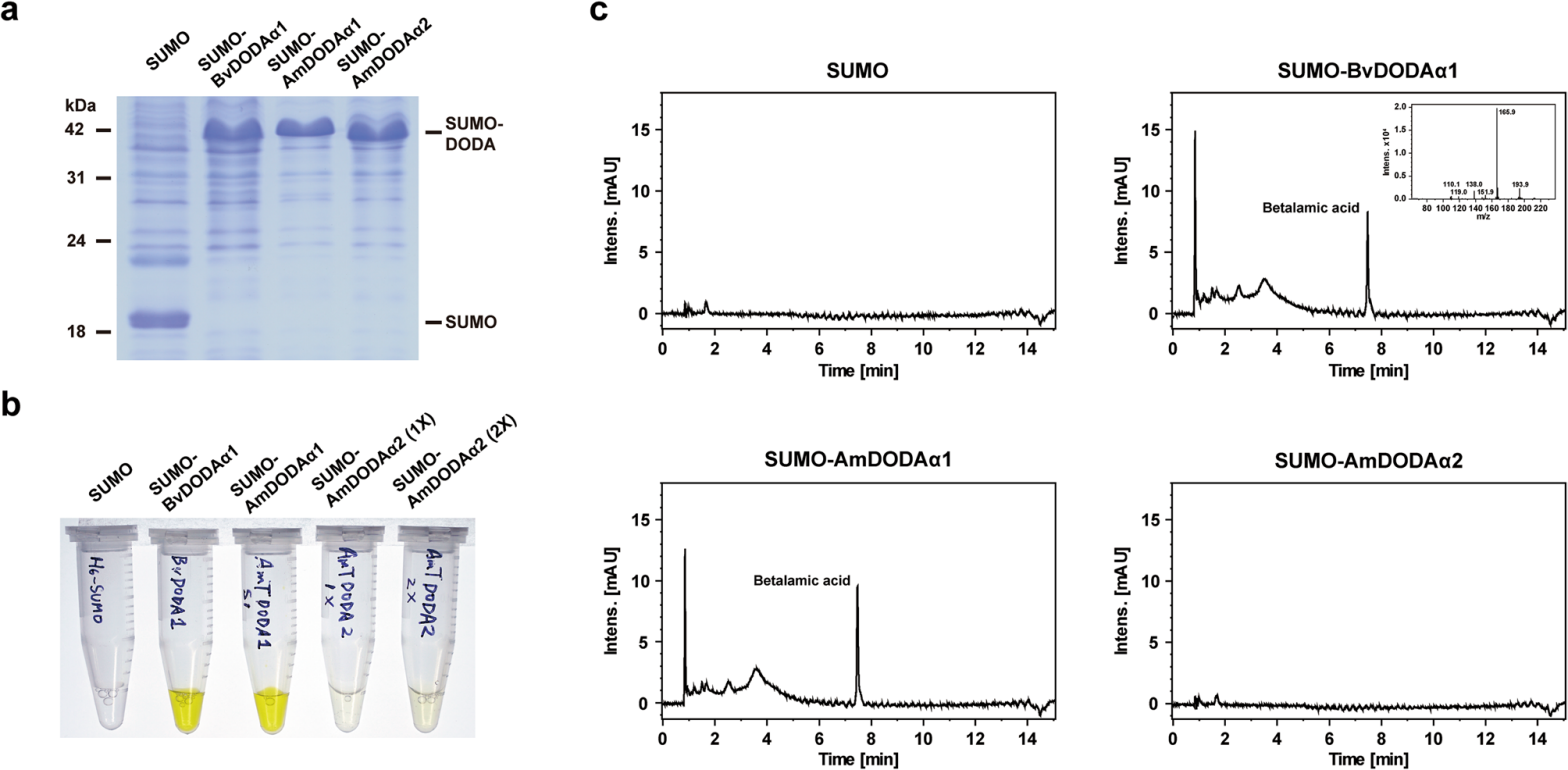
Examination of the L-DOPA 4,5-dioxygenase activity of *AmDODAα1* and *AmDODAα2* in vitro. (**a**) Crude extracts prepared from *E. coli* expressing SUMO-fused BvDODAα1, AmDODAα1, or AmDODAα2 were examined by Coomassie brilliant blue staining. Recombinant proteins were expressed in similar amounts. (**b**) Enzymatic reactions were conducted following the method described by Sasaki et al^32^ with some modifications. The reaction mixtures contained L-DOPA, ascorbic acid, and the crude extract prepared from *E. coli* harboring the *BvDODAα1*, *AmDODAα1*, or *AmDODAα2* gene. Yellow color derived from betalamic acid was examined to evaluate the enzymatic activity of L-DOPA 4,5-dioxygenase. (**c**) Elution profiles of the in vitro reactions were performed by LC–MS/MS. Betalamic acid was confirmed by a mass fragmentation profile of the peak at a retention time of 7.5 min.

To verify enzyme activity in vitro, AmDODAα1 and AmDODAα2 were expressed as SUMO-fused recombinant proteins in an *Escherichia coli* expression system (Fig. 5a). Enzymatic reactions were conducted following the method described by Sasaki et al^32^, in which crude extracts prepared from *E. coli* were used. After incubation for 5 min at 30 °C, a bright yellow color derived from betalamic acid was observed in the reaction mixture containing L-DOPA, ascorbic acid, and a crude extract prepared from *E. coli* harboring *AmDODAα1* or *BvDODAα1*, but not *AmDODAα2* (Fig. 5b). However, only a very weak yellow color was observed when the reaction mixture contained twofold crude extract prepared from *E. coli* harboring *AmDODAα2* (Fig. 5b). As a control, a reaction mixture containing the crude extract was prepared from *E. coli* harboring only the vector, and no color was observed (Fig. 5b). The reaction products were then subjected to LC–MS/MS analysis and revealed that the clear peak at a retention time of 7.5 min was betalamic acid (Fig. 5c). These results confirm that AmDODAα2 exhibits marginal levels of L-DOPA 4,5-dioxygenase activity.

### Reconstruction of the core betalain biosynthesis pathway of *A. tricolor* in *N. benthamiana*

In this study, we also attempted to use TRV-based virus-induced gene silencing (VIGS) to examine the functional activities of genes involved in betalain biosynthesis in *A. tricolor*. However, the transient silencing of *AmCYP76AD1* in *A. tricolor* was particularly challenging and failed in our hands. In addition, the attempted overexpression of *AmCYP76AD1* to complement the betalain pigments in the leaves of AMG was unsuccessful using an agroinfiltration system. These differences might have resulted from the different varieties and low transformation efficiency of *A. tricolor*^33^.

**Figure 6:**
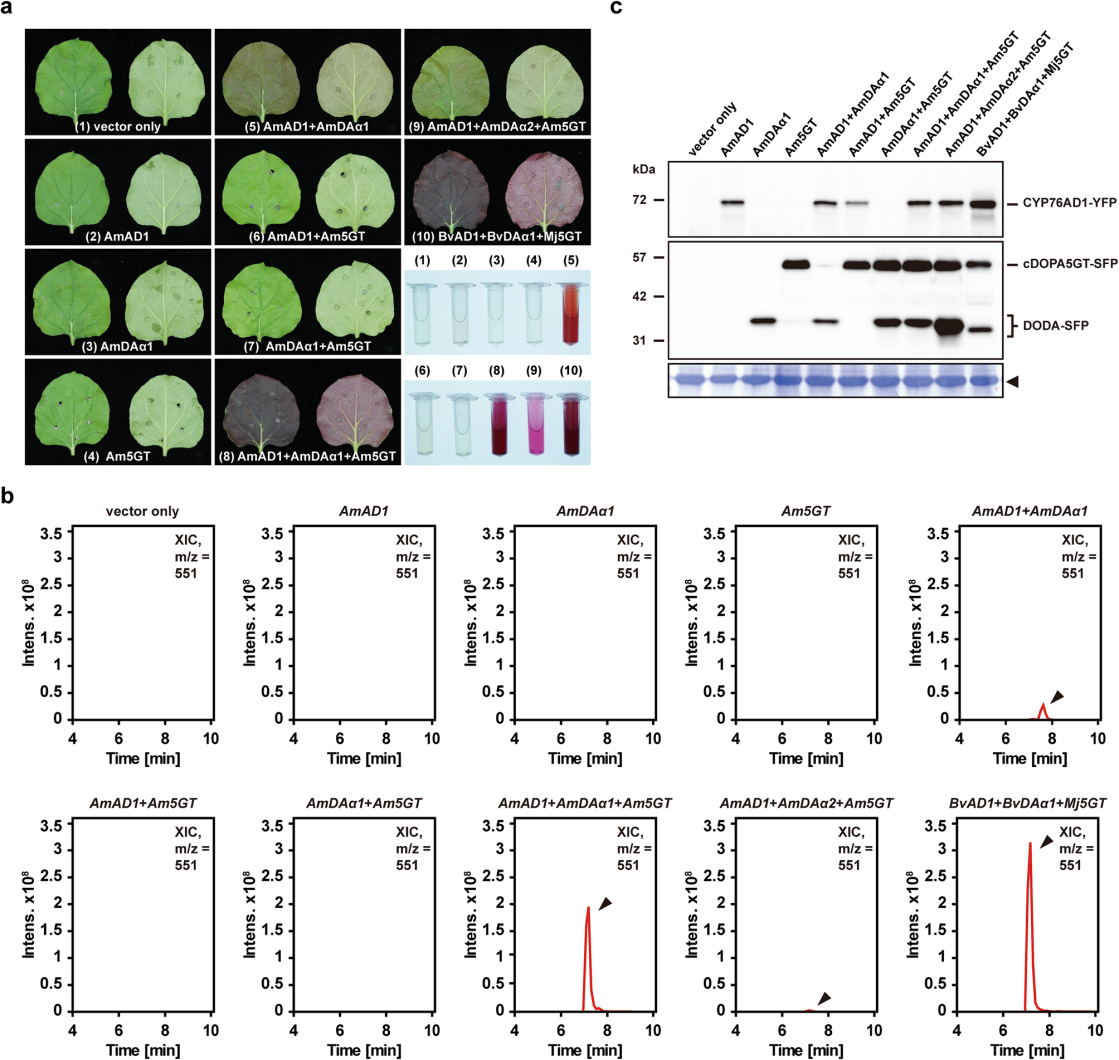
Reconstruction of the core betalain biosynthesis pathway of *A. tricolor* in *N. benthamiana* by agroinfiltration for the heterologous engineering of betalain pigments. (**a**) *N. benthamiana* leaves coinfiltrated with *Agrobacterium* harboring plasmids for the expression of *BvCYP76AD1-YFP* (*BvAD1*), *BvDODAα1-SFP* (*BvDAα1*), *MjcDOPA5GT-SFP* (*Mj5GT*), *AmCYP76AD1-YFP* (*AmAD1*), *AmDODAα1-SFP* (*AmDAα1*), *AmDODAα2-SFP* (*AmDAα2*), and *AmcDOPA5GT-SFP* (*Am5GT*). Both the adaxial (left) and abaxial (right) sides of leaves are presented in each panel. Bottom right corner indicated the betalain pigments produced in *N. benthamiana*. (**b**) Extracted betalain pigments were examined for betanin content by LC–MS/MS analysis. Shown are XICs of masses corresponding to betanin (m/z = 551). Time, retention time (min). (**c**) Western blotting assays were conducted to examine the expression levels of YFP-tagged CYP76AD1 (upper panel), SFP-tagged DODA (middle panel), and SFP-tagged cDOPA5GT (middle panel) using antibodies against the YFP- or FLAG (SFP)-tag. As a loading control, the large subunit of Rubisco visualized with Coomassie brilliant blue staining is indicated by the arrowhead (lower panel).

To reconstruct the core betalain biosynthesis pathway of *A. tricolor*, *AmCYP76AD1*, *AmDODAα1*, and *AmcDOPA5GT* were transiently overexpressed in *N. benthamiana* leaves by agroinfiltration for the heterologous engineering of betalain pigments. Similar to the vector-only control, the heterologous expression of single *AmCYP76AD1*, *AmDODAα1*, or *AmcDOPA5GT* was not sufficient to produce any betalain pigment in *N. benthamiana* (Fig. 6a). However, low production of betalain pigments was observed when *AmCYP76AD1* and *AmDODAα1* were coexpressed in *N. benthamiana* (Fig. 6a). In contrast, no betalain pigment was observed when *AmCYP76AD1* and *AmcDOPA5GT* or *AmDODAα1* and *AmcDOPA5GT* were coexpressed in *N. benthamiana* (Fig. 6a). Only the coexpression of *AmCYP76AD1*, *AmDODAα1*, and *AmcDOPA5GT* together was sufficient to produce high amounts of betalain pigments in *N. benthamiana*, which resulted in a strong red-violet color (Fig. 6a). The strong red-violet color was similar to that in the positive control in which *BvCYP76AD1*, *BvDODAα1*, and *MjcDOPA5GT* were coexpressed in *N. benthamiana* (Fig. 6a). As expected, the coexpression of *AmCYP76AD1*, *AmDODAα2*, and *AmcDOPA5GT* only produced marginal levels of betalain pigments, which were barely detectable (Fig. 6a). Consistently, high production of betanin was observed only when *AmCYP76AD1*, *AmDODAα1*, and *AmcDOPA5GT* were coexpressed in *N. benthamiana* leaves (Fig. 6b). Together with the comparable amount of proteins detected by western blotting (Fig. 6c, Supplementary Fig. S5), our results suggest that the enzyme activities of AmCYP76AD1, AmDODAα1, and AmDOPA5GT are sufficient to construct the core betalain biosynthesis pathway of *A. tricolor*.

## Discussion

Molecular genetics have shed light on the betalain biosynthesis pathway and its evolutionary significance in Caryophyllales. Based on phylogenetic analysis, CYP76AD homologues can be classified into α, β, and γ clades^9^. To date, only the functions of CYP76ADα and CYP76ADβ clade homologues, such as *CYP76AD1* and *CYP76AD6*, have been reported^10^. For example, the cosilencing of *CYP76AD1* and *CYP76AD6* represses the production of betacyanins and betaxanthins in *B. vulgaris*, causing a green leaf phenotype^16^. In this study, a *CYP76AD6*-like (*AmCYP76AD6*) gene, belonging to the CYP76ADβ clade according to phylogenetic construction and LOGO analysis (Fig. 2c,d), was also identified in *A. tricolor* through transcriptome analysis (Supplementary Fig. S6). However, the expression of *AmCYP76AD6* was extremely low and was difficult to detect in AMR and AMG. As a result, it is difficult to functionally connect *AmCYP76AD6* with the production of betalains in *A. tricolor*. In addition, although *PPO*, a polyphenol oxidase gene, and *CATPO*, a catalase-phenol oxidase gene, were previously proposed to be involved in betalain biosynthesis via monophenolase activity^34,35^, their transcripts did not show highly differential expression patterns between AMR and AMG (Fig. 1d, Supplementary Fig. S1c). As a result, we propose that the elevated expression of *AmCYP76AD1* is necessary for the occurrence of a red-violet color phenotype in *A. tricolor*; in contrast, the loss of *AmCYP76AD1* expression results in a green color phenotype in *A. tricolor* (Fig. 1a–d). The existence of the *AmCYP76AD1* gene in AMG examined by PCR using genomic DNA as a template confirmed the loss of *AmCYP76AD1* expression in AMG (Supplementary Fig. S7). Together with the functional characterization of the enzymatic activity of AmCYP76AD1 through the heterologous engineering of betalain pigments in *N. benthamiana* (Figs. 3b, 6a), we conclude that AmCYP76AD1, a CYP76ADα homologue required for the initiation of the betalain biosynthesis pathway, plays a key role in betalain pigment accumulation in *A. tricolor*. Accordingly, *AmCYP76AD1* displayed higher transcript levels in the upper leaves of AMR, which contained higher content of betalains than those in the lower leaves of AMR (Fig. 2a,b, Supplementary Fig. S2).

In recent years, with the elucidation of the central committed steps of the betalain biosynthesis pathway, comparative transcriptome analyses have been intensively applied to identify genes involved in regulating betalain biosynthesis in Caryophyllales^16,20,30,36,37^. However, numerous duplication events have led to difficulty in elucidating the functional activities of key enzymes in betalain-pigmented species through annotation^10^. For example, duplication events gave rise to two major clades of DODA homologues, DODAα and DODAβ, but only one gene paralog in the DODAα clade of each species exhibits high levels of L-DOPA 4,5-dioxygenase activity^12,21^. Thus, it is necessary to examine the possible involvement of annotated genes in betalain biosynthesis on the basis of experimental evidence. In this study, the *AmDODAα1* and *AmDODAα2* genes, which belong to the DODAα clade according to phylogenetic construction and LOGO analysis (Fig. 4b,c), were identified in *A. tricolor* through transcriptome analysis (Supplementary Fig. S4, Table S4). Based on the heterologous engineering of betalain pigments in *N. benthamiana* and in vitro biochemical studies (Figs. 3b, 5b), we report that AmDODAα1 displayed a high level of L-DOPA 4,5-dioxygenase activity to produce betalamic acid, but such activity was barely detectable for AmDODAα2. These results indicate that at least one duplication event has occurred in the DODAα lineage of *A. tricolor*, and the primary function of AmDODAα2 remains to be further studied.

Betalains are composed of betacyanins and betaxanthins. In contrast to betaxanthins, which are derived from betalamic acid via spontaneous condensation with amino acids or other amines, a large number of betacyanins are composed of betanidin conjugated with glycosyl moieties^9,10^. We characterized the function of *AmcDOPA5GT*, a *cyclo*-DOPA 5-*O*-glucosyltransferase gene, through the heterologous engineering of betalain pigments in *N. benthamiana*. The coexpression of *AmCYP76AD1*, *AmDODAα1*, and *AmcDOPA5GT* enabled the production of high levels of betalain pigments with a dark red color (Fig. 6a). In contrast, low production of betalain pigments was observed when *AmCYP76AD1* and *AmDODAα1* were coexpressed (Fig. 6a). Our results suggest the importance of *AmcDOPA5GT* in the glycosylation reaction during betalain biosynthesis in *A. tricolor*. In fact, the metabolic pathway of betalain biosynthesis is very complex due to multiple glycosylation steps, and different betacyanins have been identified^10,38^. For example, betanin, the most common betacyanin, is not only produced by *cyclo*-DOPA 5-*O*-glucosyltransferase but is also produced by betanidin 5-*O*-glucosyl-transferase through the glycosylation of betanidin^39,40^. In this study, *AmB5GT*, a betanidin 5-*O*-glucosyl-transferase gene, was also identified through comparative transcriptome analyses (Supplementary Table S4). Although *AmcDOPA5GT* showed higher expression levels than *AmB5GT* in both AMR and AMG (Supplementary Table S4), it remains to be determined which of the two glycosylation routes is more important for the formation of betanin in *A. tricolor*.

Recently, betalain biosynthesis in different pitaya species, such as *Hylocereus polyrhizus*, *Hylocereus costaricensis*, *Hylocereus undatus*, and *Hylocereus megalanthus*, has been intensively studied through comparative transcriptome analysis^36,37,41,42^. However, further studies remain to be conducted to provide experimental evidence and strengthen the understanding of the roles of candidate genes in betalain biosynthesis. Here, complementation assays conducted through the heterologous engineering of betalain pigments in nonbetalain-producing plants provided a solution for the easy and rapid comparison of the functional activities of genes involved in the core betalain biosynthesis pathway between betalain-pigmented species of Caryophyllales. Using the coexpression of *BvCYP76AD1*, *BvDODAα1*, and *MjcDOPA5GT* in *N. benthamiana* as a positive control, the functional activities of *A. tricolor* genes responsible for betalain synthesis could be compared through a series of complementation assays (Fig. 3b–d). We showed that comparable amounts of betalain pigments were observed when the functional activities of positive genes were individually replaced with *AmCYP76AD1*, *AmDODAα1*, and *AmcDOPA5GT* in transient coexpression assays (Fig. 3b–d). Our results indicate that *AmCYP76AD1*, *AmDODAα1*, and *AmcDOPA5GT* exhibit high tyrosinase, L-DOPA 4,5-dioxygenase, and *cyclo*-DOPA 5-*O*-glucosyltransferase activities, respectively, which are similar to those in *B. vulgaris* and *M. jalap*a. Accordingly, in vitro biochemical studies demonstrated that AmDODAα1 displayed comparable L-DOPA 4,5-dioxygenase activity to BvDODAα1 in producing betalamic acid (Fig. 5b). These results provide novel insights into betalain biosynthesis and evolution in *A. tricolor*.

In conclusion, a comparative transcriptome analysis combined with functional and enzymatic studies were performed to reveal the core betalain biosynthesis pathway of *A. tricolor.* The heterologous engineering of betalain pigments through the coexpression of *AmCYP76AD1*, *AmDODAα1*, and *AmcDOPA5GT* in *N. benthamiana* enabled the production of high amounts of betalain pigments with a red-violet color similar to those in the red-leaf cultivar of *A. tricolor*. Although the metabolic pathway of betalain biosynthesis is very complex, the core betalain biosynthesis pathway of *A. tricolor* constructed here not only provides a basal framework for examining genes related to betalain biosynthesis within the species of *Amaranthaceae* but also sheds light on the evolution of the betalain biosynthesis pathway in Caryophyllales.

## Methods

### Plant materials and growth conditions

*A. tricolor*, *B. vulgaris*, *M. jalapa*, and *N. benthamiana* plants were grown at 26 °C in a semicontrolled walk-in chamber under a 16:8-h light:dark photoperiod. Soil (Jiffy) mixed with vermiculite and pearlstone was used. Seeds of *A. tricolor* cv. Hung Hsien (red-leaf cultivar) and *A. tricolor* cv. Pai Hsien (green-leaf cultivar) were purchased from KNOWN-YOU SEED CO., LTD.

### Betalain pigment extraction and measurement

For betalain pigment measurement, betalain contents were determined as described previously with some modification^43^. Briefly, leaves of seedlings were collected and ground into powder in liquid nitrogen. Betalain pigments were extracted with extraction solution (methanol:chloroform:H_2_O [1:2:1]). After centrifugation, the upper (hydrophilic) layer was collected to measure the absorbance at 538 nm and 476 nm for betacyanins and betaxanthins, respectively. The relative betalain content was calculated with the following equation: (*A*_538_ + *A*_476_)/gram).

### Plasmid construction

All plasmid constructs were generated using standard restriction site reconstruction methods and confirmed by DNA sequencing. *AmCYP76AD1*, *AmDODAα1*, *AmDODAα2*, *AmcDOPA5GT*, *BvCYP76AD1*, *BvDODAα1*, and *MjcDOPA5GT* were amplified from *A. tricolor*, *B. vulgaris*, or *M. jalapa* cDNA libraries using AccuPrime pfx DNA polymerase (Invitrogen). For the transient expression of C-terminal YFP- or FLAG (SFP)-tagged proteins in *N. benthamiana*, PCR products encoding *AmCYP76AD1*, *AmDODAα1*, *AmDODAα2*, *AmcDOPA5GT*, *BvCYP76AD1*, *BvDODAα1*, and *MjcDOPA5GT* were subcloned into pBA-C-SFP or pBA-C-YFP vectors under the control of a *Cauliflower mosaic virus* (*CaMV*) *35S* promoter^44^. To produce N-terminal SUMO-tagged recombinant proteins, PCR products encoding *AmDODAα1*, *AmDODAα2*, and *BvDODAα1* were subcloned into the pET-SUMO (Invitrogen) vector^45^. For the VIGS assay, a cDNA fragment of *AmCYP76AD1* was amplified and subcloned into the pTRV2 vector^46^. The primer sequences used for plasmid construction are listed in Supplementary Table S5.

### Quantitative real-time polymerase chain reaction (qRT-PCR) and statistical analysis

TRIzol™ (Invitrogen)-extracted total RNA was reverse transcribed using SuperScript III First-Strand Synthesis SuperMix (Invitrogen) according to the manufacturer’s instructions. Briefly, each sample was prepared from the leaves of three biologically distinct 3-week-old or 4-week-old *A. tricolor* plants. Then, cDNA was synthesized from 1 μg of total RNA using a mixture of random hexamers and oligo(dT)_20_ under the following conditions: 25 °C for 10 min, followed by 50 °C for 40 min. The cDNA was employed as a template for qRT-PCR using the KAPA SYBR Fast qPCR Kit (Kapa Biosystems). Three technical replicates were performed on a CFX96™ Real-time System (Bio-Rad) under the following conditions: 95 °C for 3 min, followed by 40 cycles of 95 °C for 10 s and 55 °C for 30 s. The expression levels of selected genes were determined by normalization to the reference gene *Actin*. Statistically significant differences were determined using Student’s *t*-test in SPSS version 20.0. The primer sequences employed for qRT-PCR analyses are listed in Supplementary Table S1. PCR analyses using genomic DNA extracted from AMR and AMG as a template were performed to confirm the specificity of the primers (Supplementary Fig. S7).

### Transient coexpression assay and western blotting

Plasmids for the transient expression of AmCYP76AD1-YFP, AmDODAα1-SFP, AmDODAα2-SFP, AmcDOPA5GT-SFP, BvCYP76AD1-YFP, BvDODAα1-SFP, or MjcDOPA5GT-SFP were transformed into the *Agrobacterium tumefaciens* strain ABI. C-terminal tagged proteins were coexpressed using a mixture of *A. tumefaciens* carrying the desired constructs in *N. benthamiana* leaves by agroinfiltration following the method described previously^47^. After three days, the infiltrated leaves were photographed and ground into a powder in liquid nitrogen for total cell extract preparation. Briefly, 0.1 g of sample powder was added to 0.2 ml of 2.5 × SDS sample buffer (5 mM EDTA, 5% SDS, 0.3 M Tris–HCl, pH 6.8, 20% glycerol, 1% β-mercaptoethanol, and bromophenol blue), which was then heated at 95 °C in a dry bath for 10 min. After centrifugation at 13,000× *g* for 10 min, the supernatant was obtained, and total proteins were separated by SDS-PAGE. Western blotting assays were performed to monitor protein levels using specific polyclonal and monoclonal antibodies against YFP- and FLAG-tag, respectively. Chemiluminescence signals generated by ECL reagents (PerkinElmer) were captured with an ImageQuant LAS 4000 mini imager (GE Healthcare). All experiments were repeated at least three times using biologically distinct samples prepared from two infiltrated leaves.

### In vitro L-DOPA 4,5-dioxygenase activity assay and liquid chromatography-tandem mass spectrometry (LC–MS/MS) analysis

An in vitro L-DOPA 4,5-dioxygenase activity assay was performed according to the method described previously with some modifications^32^. Briefly, plasmids for the expression of N-terminal SUMO-tagged *AmDODAα1*, *AmDODAα2* and *BvDODAα1* were transformed into *Escherichia coli* strain BL21 (DE3). The transformants were grown in 50 ml LB medium, and the recombinant proteins were induced with 0.2 mM IPTG at 22 °C for 16 h. Harvested cells were washed, resuspended, and disrupted by sonication in 50 mM sodium phosphate buffer (pH 7.0). The crude extract (supernatant) was used for the enzyme activity assay after centrifugation at 14,000× *g* for 15 min. The amount of recombinant protein was quantified with Protein Assay Reagent (Bio-Rad) and via Coomassie blue staining SDS-PAGE with BSA as the standard. Basically, the reaction (100 μl) was performed with the crude extract containing 8 μg DODA protein, 27 mM ascorbic acid, and 6.75 mM L-DOPA at 30 °C for 5 min.

LC–MS/MS was performed using a Dionex UltiMate 3000 system (Thermo Fisher Scientific) linked with an amaZon speed-ion trap mass spectrometer (Bruker). Betalamic acid was detected on a Waters BEH shield RP18 column with two eluting solvent systems: (A) H_2_O with 0.1% formic acid, (B) 100% acetonitrile. The gradient elution program was set as follows: 0–3 min (100% A), 9 min (55% A and 45% B), 12–13 min (100% B). The flow rate was 0.3 ml min^-1^, and the detector wavelength was 424 nm. The electrospray ionization mass parameters were set as follows: 4.5 kV capillary, 500 V end plate offset voltage, 40.0 psi nebulizer pressure, 8.0 l min^−1^ dry gas, and 230 °C dry temperature. The measurement was operated in multiple reaction-monitoring (MRM) with the positive ion mode. The MRM was set 182 → 165 m/z to detect tyrosine, 198 → 181 m/z to detect *L*-DOPA, 212 → 166 m/z to detect betalamic acid, 389 → 345 m/z to detect betanidin, and 551 → 389 m/z to detect betanin.

### Next-generation sequencing and MA plot

To perform next-generation sequencing, aerial tissues derived from three biologically distinct 3-week-old *A. tricolor* plants were collected. Total RNA was extracted using the RNeasy Plant Mini Kit (Qiagen) according to the manufacturer’s instructions. RNA quality was examined via 1.2% (wt/vol) formaldehyde gel electrophoresis and with an Experion RNA analysis kit (Bio-Rad, Munich). Only high-quality RNA was used for next-generation sequencing performed on the Illumina HiSeq 4000 platform with 150 paired-end reads. For each dataset (AMR and AMG), 100 million reads were generated, and de novo assembly was performed with the Trinity tool. The assembled transcripts were annotated with BlastX in UniProt. Gene expression levels were normalized as FPKM values, and differentially expressed genes were identified according to an FDR < 0.05 and logFC > 2 or < −2 (Supplementary Tables S2, S3). An MA plot was generated based on the average concentration (logCPM) and fold-change (logFC) values to show the relative abundances of transcripts between AMR and AMG.

### Phylogenetic tree reconstruction and LOGO analysis

Phylogenetic trees were reconstructed using MEGA-X software based on the protein sequence comparisons of CYP76AD and DODA homologues from different betalain-producing species. Multiple sequence alignments were performed using the MUSCLE program and were processed to generate a maximum likelihood phylogenetic tree via the Jones–Taylor–Thornton (JTT) model with bootstrapping to perform molecular evolutionary analysis. The numbers at the branch points are bootstrap values representing the percentages of replicate trees based on 1000 repeats. LOGO analyses were performed via WebLogo (http://weblogo.berkeley.edu/logo.cgi) based on selected conserved amino acids of CYP76AD and DODA homologues reported previously^9,12,21,48^. The species, families, and accession numbers of CYP76AD and DODAα homologues are available in Supplementary Table S6.

## Supplementary Information

Supplementary Information 1.

Supplementary Information 2.

Supplementary Information 3.

Supplementary Information 4.

Supplementary Information 5.

Supplementary Information 6.

Supplementary Information 7.

## Data Availability

Sequencing data generated for this study are deposited at Short Read Archive with the accession code SRR15044103.
