# Author Correction: Elucidation of the core betalain biosynthesis pathway in *Amaranthus tricolor*

**DOI:** 10.1038/s41598-021-94665-9

**Published:** 2021-07-27

**Authors:** Yu-Cheng Chang, Yi-Ching Chiu, Nai-Wen Tsao, Yuan-Lin Chou, Choon-Meng Tan, Yi-Hsuan Chiang, Pei-Chi Liao, Ya-Chien Lee, Li-Ching Hsieh, Sheng-Yang Wang, Jun-Yi Yang

**Affiliations:** 1 Institute of Biochemistry, National Chung Hsing University, 145 Xingda Road, Taichung, 40227 Taiwan; 2 Department of Forestry, National Chung Hsing University, Taichung, 402 Taiwan; 3 Institute of Genomics and Bioinformatics, National Chung Hsing University, Taichung, 402 Taiwan; 4 Institute of Biotechnology, National Chung Hsing University, Taichung, 402 Taiwan; 5 Advanced Plant Biotechnology Center, National Chung Hsing University, Taichung, 402 Taiwan

Correction to: *Scientific Reports* https://doi.org/10.1038/s41598-021-85486-x, published online 17 March 2021

The original version of this Article contained errors.

A Data Availability section was omitted. It is now included and reads:

Sequencing data generated for this study are deposited at Short Read Archive with the accession code SRR15044103.

Additionally, one of the images in Figure 1D was missing an axis label. This figure has been updated. The original Figure 1 and accompanying legend appear below.

**Figure 1:**
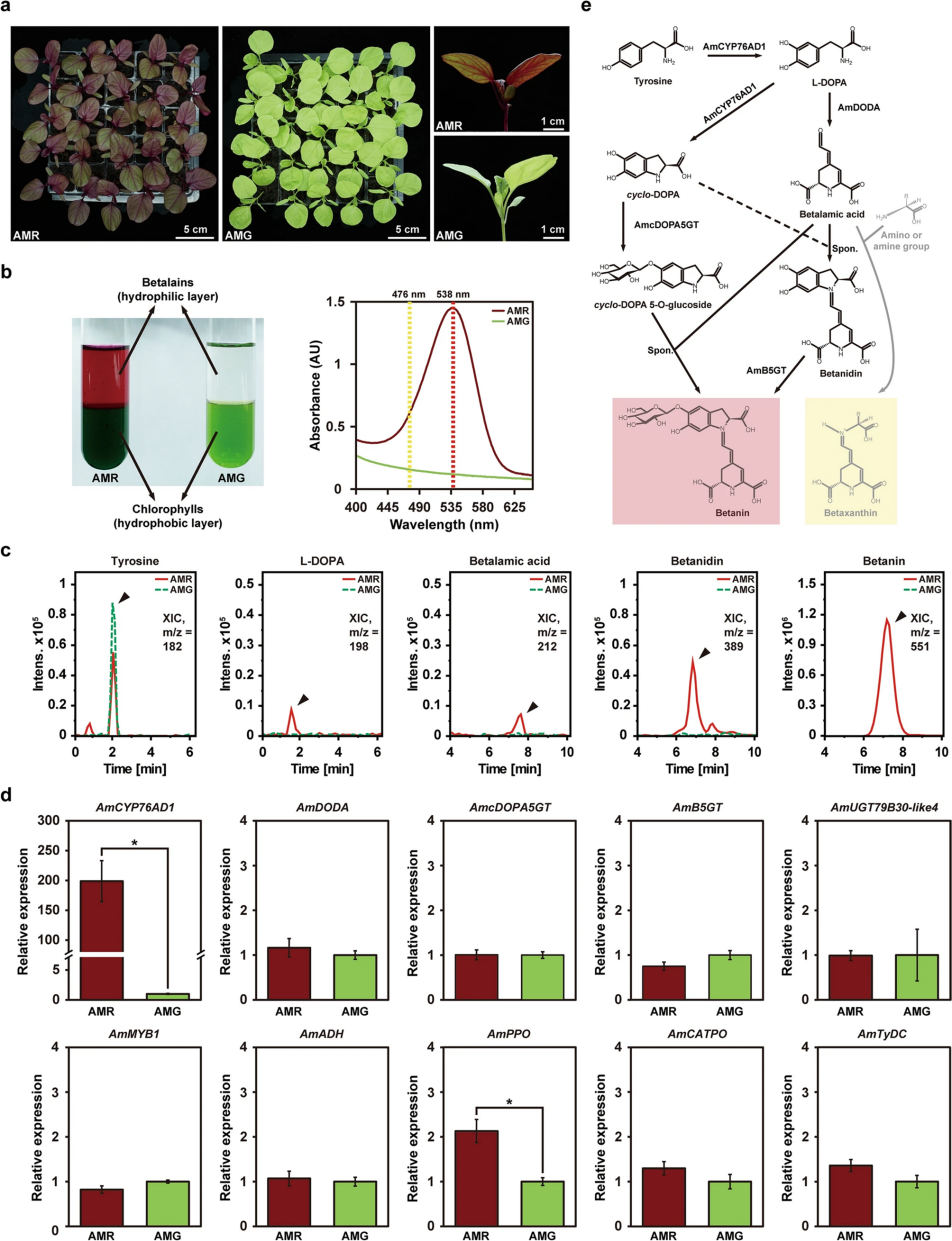
Identification of *AmCYP76AD1* as a key element required for betalain pigment production in *Amaranthus tricolor*. (**a**) The leaf-color phenotypes of the red-leaf cultivar (AMR) and green-leaf cultivar (AMG) of three-week-old *A. tricolor*. (**b**) Extraction of chlorophyll pigments (hydrophobic layer) and betalain pigments (hydrophilic layer) from three-week-old leaves of AMR and AMG (left panel). Absorbance spectra of the extracted betalain pigments from AMR and AMG (right panel). The absorbance at 538 nm for betacyanins is indicated with a red dashed line, and the absorbance at 476 nm for betaxanthins is indicated with a yellow dashed line. (**c**) Liquid chromatography-tandem mass spectrometry (LC–MS/MS) analysis of three-week-old leaves of AMR and AMG. Shown are extracted ion chromatograms (XICs) of masses corresponding to tyrosine (m/z = 182), *L*-DOPA (m/z = 198), betalamic acid (m/z = 212), betanidin (m/z = 389), and betanin (m/z = 551). Time, retention time (min). (**d**) Expression levels of genes related to the betalain biosynthesis pathway in AMR and AMG analyzed by qRT-PCR. Statistically significant differences were determined using Student’s *t*-test (*P < 0.01 for AMR vs. AMG). (**e**) Putative core betalain biosynthesis pathway in *A. tricolor*. *Am*, *Amaranthus tricolor*; *CYP76AD1*, *cytochrome P450 76AD1*; *DODA*, *DOPA-4,5-dioxygenase*; *cDOPA5GT*, *cyclo-DOPA 5-O-glucosyltransferase*; *B5GT*, *betanidin-5-O-glucosyltransferase*; *UGT79B30-like 4*, *UDP-glucose glucosyltransferase 79B30-like 4*; *ADH*, *arogenate dehydrogenase*; *PPO*, *polyphenol oxidase*; *CATPO*, *catalase-phenol oxidase*; *TyDC*, *tyrosine decarboxylase*.

The original Article has now been corrected.

