# Supplementary material for: Elucidation of the core betalain biosynthesis pathway in Amaranthus tricolor
Source: Sci Rep. 2021 Mar 17;11:6086. doi: 10.1038/s41598-021-85486-x (PMC7969944; doi:10.1038/s41598-021-85486-x)
Supplement: Supplementary file 1 — Supplementary Information 1. [file 41598_2021_85486_MOESM1_ESM.pdf]

Supplementary Information

Elucidation of the Core Betalain Biosynthesis Pathway in *Amaranthus tricolor*

Yu-Cheng Chang[1], Yi-Ching Chiu[1], Nai-Wen Tsao[2], Yuan-Lin Chou[1], Choon-Meng Tan[1], Yi-Hsuan Chiang[1], Pei-Chi Liao[1], Ya-Chien Lee[1], Li-Ching Hsieh[3], Sheng-Yang Wang[2] and Jun-Yi Yang[1,4,5,*]

[1]Institute of Biochemistry, National Chung Hsing University, Taichung 402, Taiwan

[2]Department of Forestry, National Chung Hsing University, Taichung 402, Taiwan

[3]Institute of Genomics and Bioinformatics, National Chung Hsing University, Taichung 402, Taiwan

[4]Institute of Biotechnology, National Chung Hsing University, Taichung 402, Taiwan

[5]Advanced Plant Biotechnology Center, National Chung Hsing University, Taichung 402, Taiwan

Corresponding author: J.-Y. Yang

Institute of Biochemistry, National Chung Hsing University, 145 Xingda Road, Taichung 40227, Taiwan

886-4-22840468 ext. 232 (Tel.); 886-4-22853487 (Fax)

jyang@nchu.edu.tw

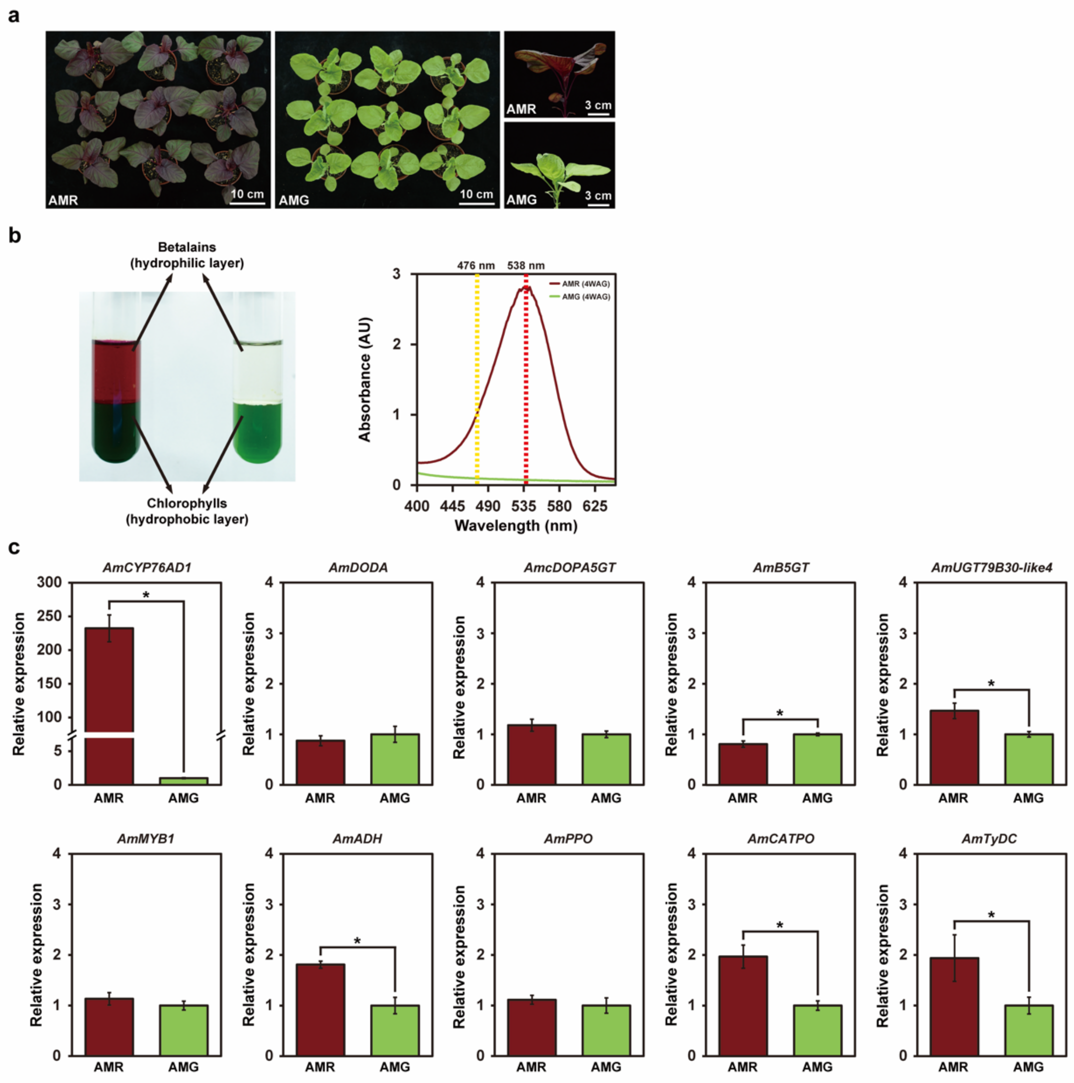

Supplementary Fig. S1 Identification of *AmCYP76AD1* as a key element required for betalain pigment production in *Amaranthus tricolor*. (**a**) The leaf-color phenotypes of the red-leaf cultivar (AMR) and green-leaf cultivar (AMG) in four-week-old *A. tricolor* plants. (**b**) Extraction of chlorophyll pigments (hydrophobic layer) and betalain pigments (hydrophilic layer) from four-week-old leaves of AMR and AMG (left panel). Absorbance spectra for the extracted betalain pigments from AMR and AMG (right panel). The absorbance at 538 nm, for betacyanins, is indicated with a red dashed line, and the absorbance at 476 nm, for betaxanthins, is indicated with a yellow dashed line. (**c**) Expression levels of genes related to the betalain biosynthesis pathway in four-week-old AMR and AMG plants analyzed by qRT-PCR. Statistically significant differences were determined using Student's *t*-test (**P* < 0.01 for AMR versus AMG). *Am*, *Amaranthus tricolor*; *CYP76AD1*, *cytochrome P450 76AD1*; *DODA*, *DOPA-4,5-dioxygenase*; *cDOPA5GT*, *cyclo-DOPA 5-O-glucosyltransferase*; *B5GT*, *betanidin-5-O-glucosyltransferase*; *UGT79B30-like 4*, *UDP-glucose glucosyltransferase 79B30-like 4*; *ADH*, *arogenate dehydrogenase*; *PPO*, *polyphenol oxidase*; *CATPO*, *catalase-phenol oxidase*; *TyDC*, *tyrosine decarboxylase*.

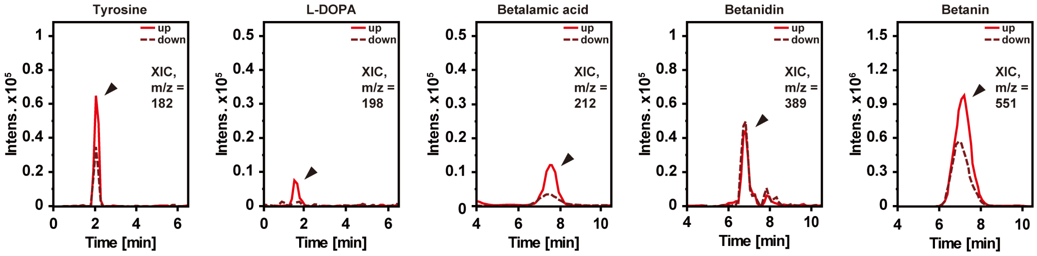

Supplementary Fig. S2 Liquid chromatography-tandem mass spectrometry (LC-MS/MS) analysis. The upper (up) and lower (down) leaves of the red-leaf cultivar (AMR) of four-week-old *A. tricolor* were collected for LC-MS/MS analyses. Shown are extracted ion chromatograms (XICs) of masses corresponding to tyrosine (m/z = 182), *L*-DOPA (m/z = 198), Betalamic acid (m/z = 212), betanidin (m/z = 389), Betanin (m/z = 551). The higher intensities of tyrosine, L-DOPA, betalamic acid, and betanin were observed in upper leaves of AMR of *A. tricolor*. Time, retention time (min).

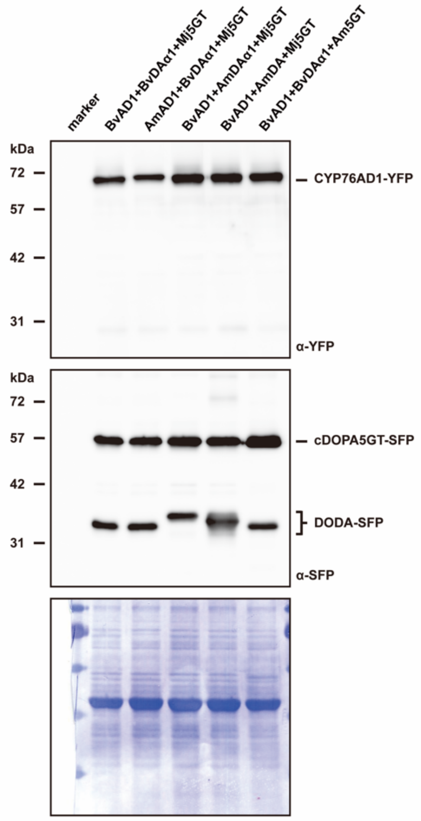

Supplementary Fig. S3 Western blotting analysis (Original images of Fig. 3d).

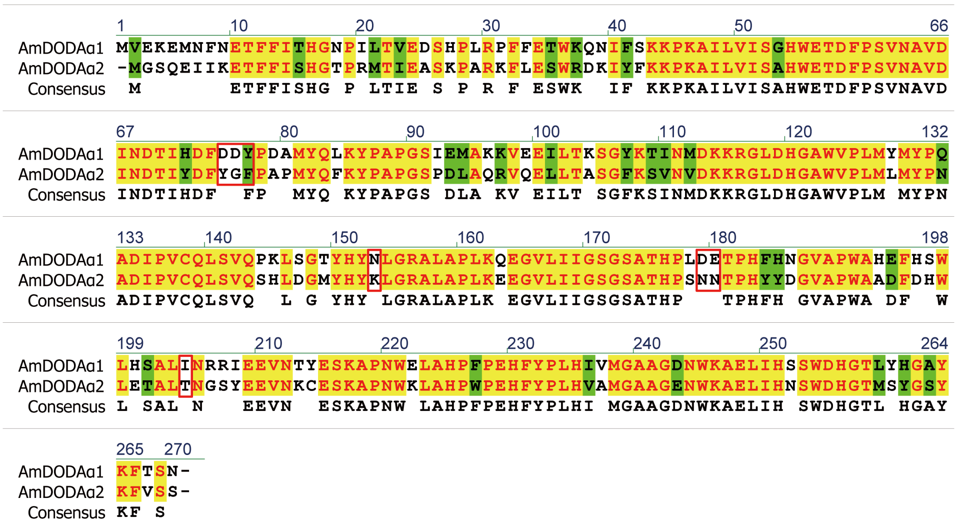

Supplementary Fig. S4 Protein sequence comparisons of AmDODAα1 and AmDODAα2. Identical residues are highlighted in yellow; similar residues are highlighted in green. The residues used for LOGO analysis are indicated with a red rectangle.

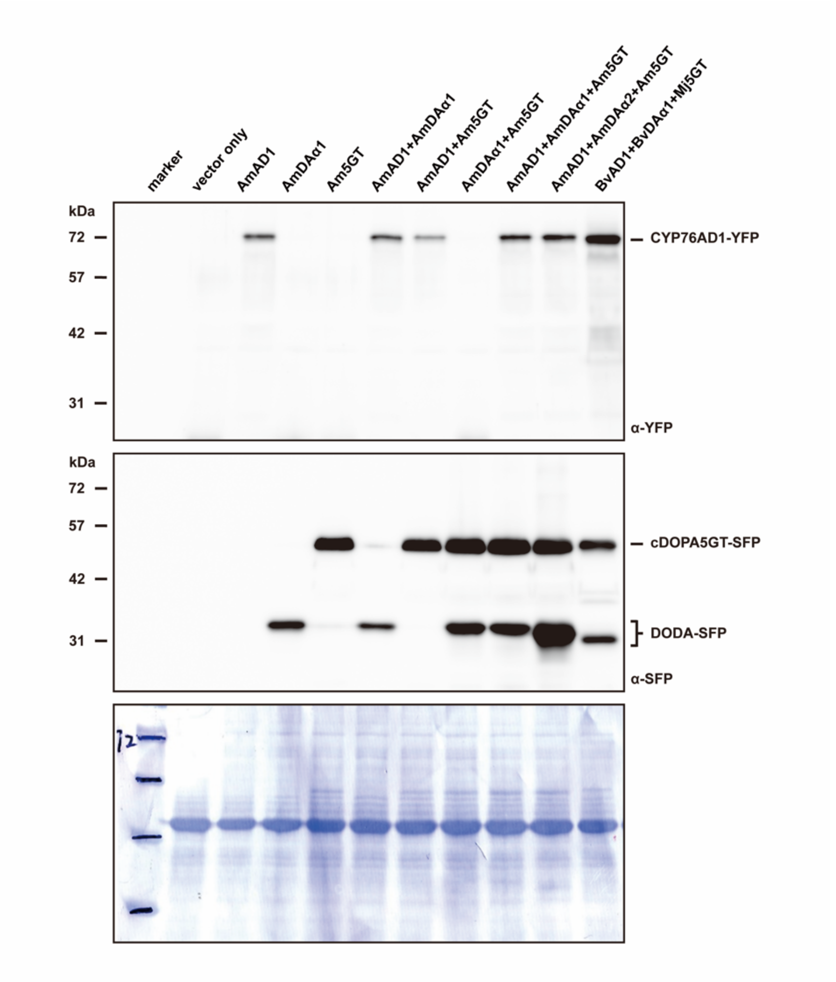

Supplementary Fig. S5 Western blotting analysis (Original images of Fig. 6c).

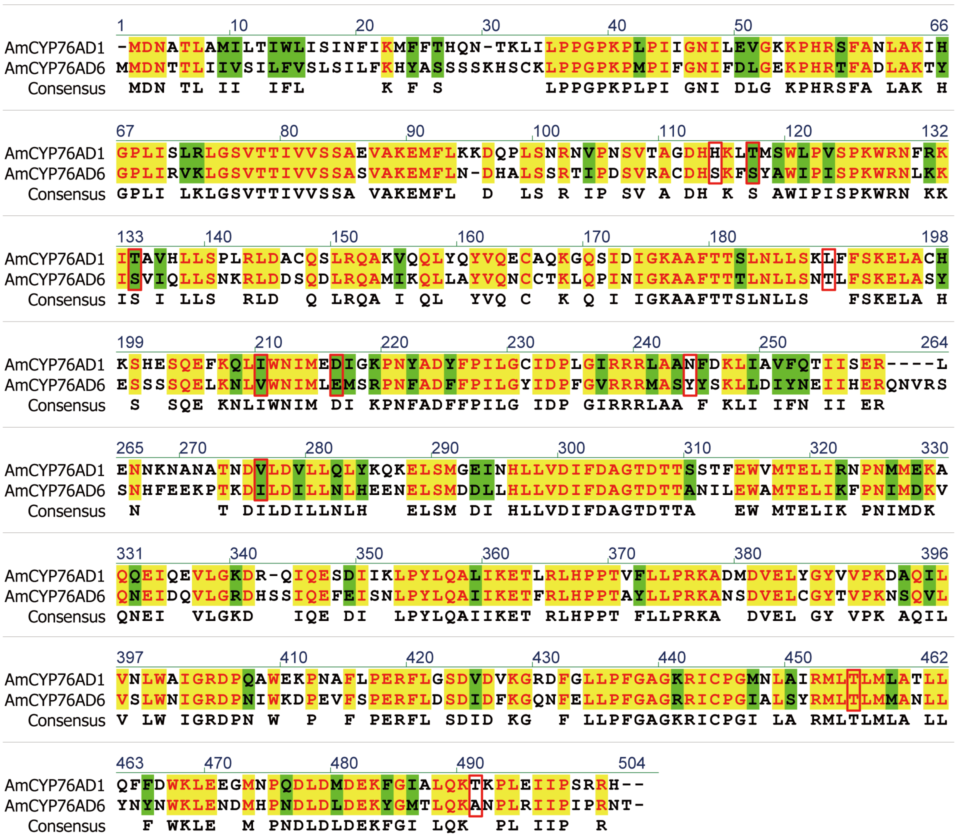

Supplementary Fig. S6 Protein sequence comparisons of AmCYP76AD1 and AmCYP76AD6. Identical residues are highlighted in yellow; similar residues are highlighted in green. The residues used for LOGO analysis are indicated with a red rectangle.

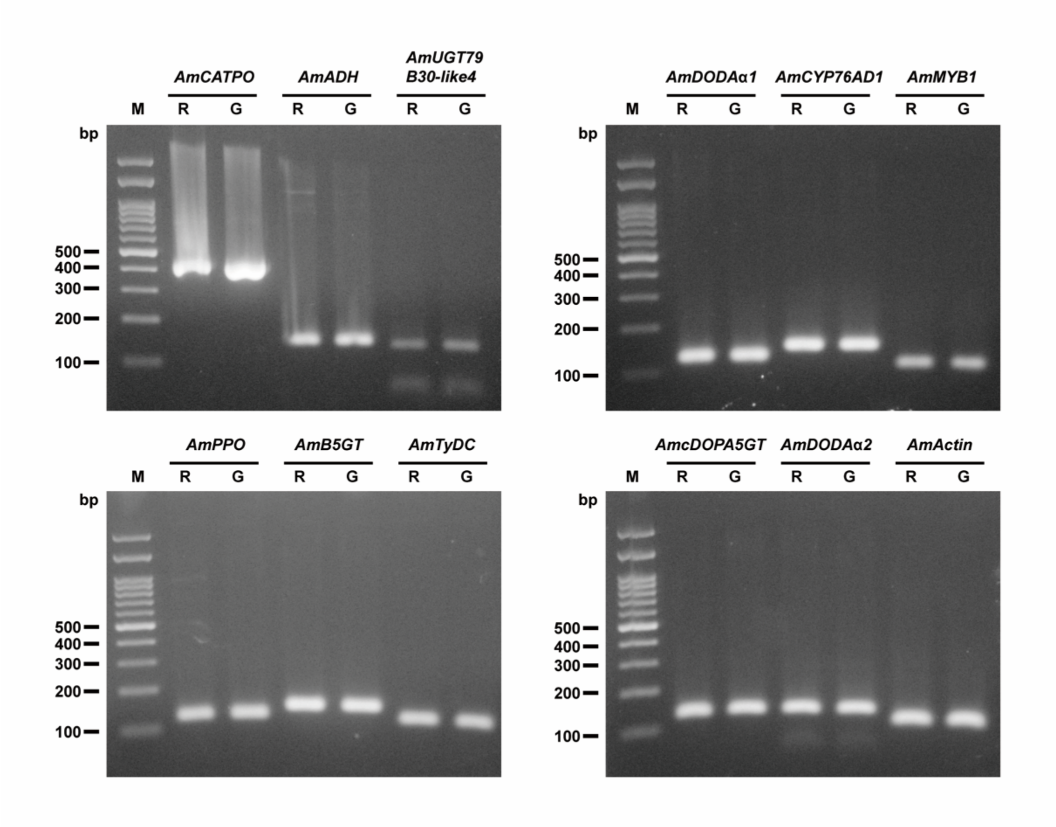

Supplementary Fig. S7 PCR analyses. Genomic DNA extracted from the red-leaf cultivar (R) and green-leaf cultivar (G) of *A. tricolor* was used as template. The primers were identical to those used for qRT-PCR analyses (Supplementary Table S1).
